# Supplementary material for: The effect of transdermal gender-affirming hormone therapy on markers of inflammation and hemostasis
Source: PLoS One. 2022 Mar 15;17(3):e0261312. doi: 10.1371/journal.pone.0261312 (PMC8923509; doi:10.1371/journal.pone.0261312)
Supplement: S1 Table — (DOCX) [file pone.0261312.s001.docx]

**Supporting information**

**S1 Table. Linear range, limit of quantification and dilution factor of inflammatory markers**

|  | Linear Range | Limit of Quantification | Dilution Factor |
| --- | --- | --- | --- |
| hs-CRP | 15.625 to 1000 pg/mL | 15.625 pg/mL | 6000x |
| α-1-antitrypsin | 1.125 to 36 ng/mL | 1.125 ng/mL | 20000x |
| TNF-α | 0.391 to 400 pg/mL | 1.5625 pg/mL | 4x |
| IFN- **ᵧ** | 0.195 to 50 pg/mL | 0.1953 pg/mL | 4x |
| IL-1b | 0.391 to 100 pg/mL | 25 pg/mL | 4x |
| IL-4 | 3.125 to 200 pg/mL | 0.7813 pg/mL | 4x |
| IL-6 | 0.293 to 300 pg/mL | 4.6875 pg/mL | 4x |
| IL-8 | 1.562 to 400 pg/mL | 6.25 pg/mL | 4x |
| IL-10 | 0.391 to 100 pg/mL | 0.3906 pg/mL | 4x |
| IL-12p70 | 0.293 to 300 pg/mL | 1.1719 pg/mL | 4x |
| IL-22 | 0.098 to 100 pg/mL | 0.3906 pg/mL | 4x |
| VCAM-1 | 15.625 to 1000 pg/mL | 15.625 pg/mL | 2600x |
| leptin | 0.092 to 4.78 ng/mL | 0.092 ng/mL | 13x |
| adiponectin | 62.5 to 1000 pg/mL | 62.5 pg/mL | 5400x |
| PF-4 | 15.625 to 500 pg/mL | 15.625 pg/mL | 16000x |
| β-thromboglobulin | 15.625 to 250 pg/mL | 15.625 pg/mL | 38000x |
| p-selectin | 125 to 2000 pg/mL | 125 pg/mL | 40x |
| fibrinogen | 0.165 to 4.444 ug/mL | 0.165 ug/mL | 1000x |
| PAI-1 | 7.8125 to 250 pg/mL | 7.8125 pg/mL | 270x |
