# Supplementary material for: The effect of transdermal gender-affirming hormone therapy on markers of inflammation and hemostasis
Source: PLoS One. 2022 Mar 15;17(3):e0261312. doi: 10.1371/journal.pone.0261312 (PMC8923509; doi:10.1371/journal.pone.0261312)
Supplement: S2 Table — (DOCX) [file pone.0261312.s002.docx]

**Supporting information**

| **Blood samples** | Visit (months) | | | | | |
| --- | --- | --- | --- | --- | --- | --- |
|  | 0 | 3 | 12 | 0 | 3 | 12 |
|  |  | | | | | |
| EDTA plasma* | 48 | 21 | 43 | 47 | 31 | 36 |
| Serum plasma** | 46 | 20 | 42 | 45 | 31 | 35 |

**S2 Table. Number of analyzed blood samples for trans women and trans men at 0, 3 and 12 months**

*Marker concentrations measured in EDTA plasma: hs-CRP, α-1-antitrypsin, TNF-α, IL-6, IL-8, IL-10, IL-22, VCAM-1, leptin, adiponectin, p-selectin, fibrinogen, PAI-1
**Marker concentrations measured in serum plasma: PF-4, β-thromboglobulin
