# Supplementary material for: The effect of transdermal gender-affirming hormone therapy on markers of inflammation and hemostasis
Source: PLoS One. 2022 Mar 15;17(3):e0261312. doi: 10.1371/journal.pone.0261312 (PMC8923509; doi:10.1371/journal.pone.0261312)
Supplement: Attachment — Submitted filename: Response to Reviewers_17-11.docx [file pone.0261312.s004.docx]

**Response to reviewers**

*We would like to thank both reviewers for the valuable suggestions on our manuscript. Below, we outline how these suggestions helped us to improve the manuscript.*

**Reviewer #1: Schutte et al looked at the effect of transdermal GAHT on markers of inflammation. The study is nicely done but the authors need to recognize the limitation of their study. The sample size is very small and no control subjects but 15 markers have been tested. This is a big limitation for the study, the authors should mention that this is an exploratory study and no way is a confirmatory study.**

*We agree that our study is exploratory and emphasized this by adding this to the abstract (p. 2):*

“In this exploratory study, 48 trans women using estradiol patches plus cyproterone acetate (CPA) and 47 trans men using testosterone gel were included.”

*We also clarified this in the introduction (p. 4):*

“As research on this topic in transgender persons is scarce, we aimed to explore the effects of GAHT on inflammation and hemostasis.”

*We also mentioned this in the discussion section (p. 18):*

“The explorative aim of our study required investigating multiple markers and multiple testing. Therefore, our results are hypothesis-generating, and not hypothesis-confirming.”

**The English used in the text is also hard to follow and needs rewriting.**

*We carefully checked the language in our entire manuscript and as a consequence, we made several improvements, especially in the Discussion section.*

**The abbreviation needs to be spelled out first time used in the body of the manuscript, for example "PAI-1" never been spelled out.**

*We thank the reviewer for noticing this, and we have now corrected this (p. 6):*

“Of the examined coagulation markers, fibrinogen is associated with cardiovascular risk (16), and plasminogen activator inhibitor-1 (PAI-1) is altered by hormone therapy in postmenopausal cis women.”

**Authors report that “Concentrations of inflammatory markers were log transformed for analysis and back transformed for presentation”. While this is probably correct, authors should verify that the residual errors from the mixed linear model have a normal distribution on the log scale by computing a normal goodness of fit statistic (for example Shapiro-Wilk test) and report that normality was verified.**

*We verified the normality of the residuals via visual inspection of the histogram and added the following to the Statistical analysis section (p. 8):*

“Normality of the residuals was verified via visual inspection of the histogram.”

**If the authors are reporting the antilog of the log scale means, this is called the geometric mean and should be labelled as such.**

*We thank the reviewer for pointing this out and now added the term ‘geometric mean’ to the captions of table 3 and figure 2a and b (p. 11 and 13).*

**Data (IL-1b, IL-4,IL12p70) failing to have a normal distribution (“heavily right skewed”-line 234) is not a good reason for omitting results (“they were not further analyzed”). Non parametric methods can be used. Medians and interquartile ranges can be reported. They can probably ignore these markers if they think they are not important markers otherwise their reason for not analyzing is not acceptable.**

*We agree with the reviewer that a heavily right skewed distribution is not a good reason for not analyzing markers. However, in the majority of samples, IL-1b, IL-4, IL-5, IL12p70 and IFN-ᵧ were undetectable. We performed analyses in which we replaced undetectable concentrations by the lower limit of detection divided by two. The residuals were normally distributed. As these analyses, performed on raw estimates of concentrations, provide unreliable results with very broad confidence intervals, we do not report them in our manuscript as we believe they do not add value to our study. They are provided below:*

*“***Analyses of inflammatory markers IL-1b, IL-4, IL-5 IL12p70 and IFN-ᵧ**

If marker concentrations were undetectable, values were replaced by the lower limit of detection divided by two.

**Trans women**

After 12 months, IL1-b changed with +199% (95% CI 8 ; 726), IL-4 changed with +37% (95% CI -30 ; 169), IL-5 changed with -14%, (95% CI -44 ; 32), IL-12p70 changed with +20% (95% CI -38 ; 134) and IFN- ᵧ changed with -24% (95% CI -57 ; 33).

**Trans men**

After 12 months, IL-1b changed with -1% (95% CI -55 ; 117), IL-4 changed with -25% (95% CI -60 ; 40), IL5 changed with -17% (95% CI -41 ; 17), IL-12p70 changed with +12% (95% CI -21 ; 60) and IFN-ᵧ changed with -6% (95% CI -39 ; 47).”

**Authors should report the sample size at each time if it is not constant (48 trans females or 47 in trans males) across time.**

*We have now added this information to the Supporting information (p. 23):*

|  | Trans women | | | Trans men | | |
| --- | --- | --- | --- | --- | --- | --- |
| **Blood samples** | Visit (months) | | | | | |
|  | 0 | 3 | 12 | 0 | 3 | 12 |
|  |  | | | | | |
| EDTA plasma* | 48 | 21 | 43 | 47 | 31 | 36 |
| Serum plasma** | 46 | 20 | 42 | 45 | 31 | 35 |

**Table 1. Number of analyzed blood samples for trans women and trans men at 0, 3 and 12 months**

*Marker concentrations measured in EDTA plasma: hs-CRP, α-1-antitrypsin, TNF-α, IL-6, IL-8, IL-10, IL-22, VCAM-1, leptin, adiponectin, p-selectin, fibrinogen, PAI-1
**Marker concentrations measured in serum plasma: PF-4, β-thromboglobulin

**There is possible selection bias if those with no baseline data were excluded. Authors should provide the flow diagram showing how many were excluded from the initial pool and why they were excluded.**

*We have now added the flow diagram showing the total number of excluded subjects, including the reasons for exclusion (p. 9):*

*.*

**Figure 1. Study flowchart**

**While the authors did not report BMI or blood pressure change adjusted results, it is not clear why this should even be considered. It would seem that change in BMI and/or blood pressure are not confounders but are another result of the hormone intervention. This needs to be clarified (especially for BP).**

*BMI and blood pressure are related to inflammation, and because we did not want to ignore potential confounders, we considered these variables for our analyses. To clarify, we added the following information to the Methods section (p. 6 and 7):*

“Secondary outcome measures were estradiol and testosterone levels, BMI and blood pressure. BMI and blood pressure are influenced by GAHT (21), and are associated with inflammation (22, 23). They were included to rule out that observed changes in concentrations of inflammatory markers were actually explained by changes in BMI or blood pressure.”

**The authors looked at 15 outcomes (Table 3). They should report nominal p values for the change from time 0 and state whether they are adjusting for multiple outcomes and multiple comparisons. The authors are commended for reporting confidence bounds but should clarify that the 95% confidence level is not adjusted for multiple outcomes.**

*We now explicitly stated that we did not control for multiple comparisons in the Statistical analysis section (p. 8):*

“No adjustment for multiple comparisons was performed.”

*We also added this information and the requested p-values to Tables 3 and 4 (p. 11 and 12):*

**Table 3. Inflammation and hemostasis markers at 0, 3 and 12 months in trans women (absolute values)**

|  | Visit (months) | | | | | |
| --- | --- | --- | --- | --- | --- | --- |
|  | 0 | 3 | | | 12 | |
| **Inflammation** |  |  | | |  | |
| *Systemic inflammatory markers* |  |  | | |  | |
| hs-CRP (µg/ml) | 0.8 (0.6, 1.1) | 0.5 (0.3, 1.0) | p = 0.036 | | 0.3 (0.2, 0.4) | p = 0.001 |
| α-1-antitrypsin (µg/ml) | 186 (173, 199) | 198 (177, 222) | | p = 0.62 | 178 (165, 192) | p = 0.24 |
| TNF-α (pg/ml) | 2.9 (2.6, 3.3) | 2.5 (2.2, 2.9) | | p = 0.057 | 2.6 (2.3, 2.9) | p = 0.021 |
| IL-6 (pg/ml) | 1.1 (0.9, 1.4) | 1.0 (0.7, 1.4) | p = 0.48 | | 0.8 (0.6, 1.0) | p = 0.012 |
| IL-8 (pg/ml) | 6.3 (5.6, 7.0) | 5.9 (5.1, 6.9) | p = 0.52 | | 5.3 (4.6, 6.1) | p = 0.024 |
| IL-10 (pg/ml) | 0.6 (0.5, 0.8) | 0.5 (0.4, 0.6) | p = 0.33 | | 0.5 (0.4, 0.6) | p = 0.056 |
| IL-22 (pg/ml) | 0.8 (0.6, 0.9) | 0.5 (0.4, 0.7) | p = 0.33 | | 0.5 (0.4, 0.7) | p = 0.056 |
| *Endothelial inflammatory markers* |  |  |  | |  | |
| VCAM-1 (ng/ml) | 382 (358, 407) | 328 (299, 360) | p = 0.001 | | 337 (315, 360) | p = 0.001 |
| *Adipose tissue markers* |  |  |  | |  | |
| Leptin (ng/ml) | 3.5 (2.4, 5.1) | 7.6 (5.1, 11.4) | p = 0.001 | | 9.8 (7.5, 12.8) | p = 0.001 |
| Adiponectin (µg/ml) | 2.3 (2.1, 2.6) | 2.6 (2.1, 3.2) | p = 0.015 | | 2.5 (2.1, 2.8) | p = 0.13 |
| **Hemostasis** |  |  |  | |  | |
| *Platelet activation markers* |  |  |  | |  | |
| Platelet factor 4 (PF-4) (µg/ml) | 2.0 (1.8, 2.3) | 1.9 (1.4, 2.4) | p = 0.28 | | 2.4 (2.2, 2.6) | p = 0.009 |
| β-thromboglobulin (µg/ml) | 5.6 (5.1, 6.1) | 5.3 (4.2, 6.5) | p = 0.35 | | 6.4 (5.8, 6.9) | p = 0.015 |
| P-selectin (ng/ml) | 36 (32, 40) | 39 (35, 43) | p = 0.29 | | 34 (30, 38) | p = 0.37 |
| *Markers of coagulation* |  |  |  | |  | |
| Fibrinogen (mg/ml) | 3.7 (3.3, 4.1) | 4.3 (3.5, 5.2) | p = 0.036 | | 3.8 (3.4, 4.1) | p = 0.78 |
| PAI-1 (ng/ml) | 23 (20, 26) | 24 (20, 30) | p = 0.46 | | 21 (18, 25) | p = 0.45 |

Predicted average marker levels (geometric mean) with 95% confidence intervals and p-values, not adjusted for multiple comparisons. Measures were log-transformed for analysis and back-transformed for presentation.

**Table 4. Inflammation and hemostasis markers at 0, 3 and 12 months in trans men (absolute values)**

|  | Visit (months) | | | | | |
| --- | --- | --- | --- | --- | --- | --- |
|  | 0 | 3 | | | 12 | |
| **Inflammation** |  |  | | |  | |
| *Systemic inflammatory markers* |  |  | | |  | |
| hs-CRP (µg/ml) | 0.3 (0.2, 0.6) | 0.5 (0.3, 0.8) | p = 0.23 | | 0.5 (0.3, 0.9) | p = 0.005 |
| α-1-antitrypsin (µg/ml) | 176 (165, 188) | 166 (150, 184) | | p = 0.099 | 166 (150, 184) | p = 0.099 |
| TNF-α (pg/ml) | 2.7 (2.4, 3.0) | 2.8 (2.5, 3.1) | | p = 0.82 | 2.8 (2.5, 3.1) | p = 0.85 |
| IL-6 (pg/ml) | 1.1 (0.9, 1.4) | 1.1 (0.8, 1.6) | p = 0.76 | | 1.0 (0.8, 1.4) | p = 0.53 |
| IL-8 (pg/ml) | 5.6 (4.8, 6.4) | 6.1 (5.4, 6.9) | p = 0.15 | | 6.7 (5.7, 7.8) | p = 0.099 |
| IL-10 (pg/ml) | 0.5 (0.4, 0.5) | 0.5 (0.4, 0.6) | p = 0.64 | | 0.5 (0.4, 0.6) | p = 0.40 |
| IL-22 (pg/ml) | 0.8 (0.6, 1.0) | 0.7 (0.5, 1.0) | p = 0.64 | | 0.8 (0.6, 1.1) | p = 0.91 |
| *Endothelial inflammatory markers* |  |  |  | |  | |
| VCAM-1 (ng/ml) | 386 (364, 410) | 407 (371, 446) | p = 0.13 | | 390 (361, 421) | p = 0.37 |
| *Adipose tissue markers* |  |  |  | |  | |
| Leptin (ng/ml) | 14.0 (10.8, 18.0) | 9.9 (7.1, 13.7) | p = 0.001 | | 6.6 (4.9, 9.1) | p = 0.001 |
| Adiponectin (µg/ml) | 3.1 (2.6, 3.6) | 2.4 (2.1, 2.9) | p = 0.001 | | 2.5 (2.1, 2.9) | p = 0.001 |
| **Hemostasis** |  |  |  | |  | |
| *Platelet activation markers* |  |  |  | |  | |
| Platelet factor 4 (PF-4) (µg/ml) | 2.1 (1.9, 2.3) | 1.7 (1.3, 2.2) | p = 0.036 | | 2.1 (1.8, 2.4) | p = 0.85 |
| β-thromboglobulin (µg/ml) | 5.4 (5.1, 5.9) | 4.4 (3.4, 5.8) | p = 0.032 | | 5.5 (5.0, 6.1) | p = 0.99 |
| P-selectin (ng/ml) | 33 (30, 37) | 36 (32, 40) | p = 0.56 | | 34 (30, 38) | p = 0.72 |
| *Markers of coagulation* |  |  |  | |  | |
| Fibrinogen (mg/ml) | 3.5 (3.2, 3.9) | 3.0 (2.3, 3.9) | p = 0.095 | | 3.2 (2.8, 3.6) | p = 0.36 |
| PAI-1 (ng/ml) | 23 (20, 25) | 26 (21, 32) | p = 0.40 | | 21 (17, 25) | p = 0.35 |

Predicted average marker levels (geometric mean) with 95% confidence intervals and p-values, not adjusted to multiple comparisons. Measures were log-transformed for analysis and back-transformed for presentation.

**Obviously, a stronger study would compare these changes to changes in a cisgender control group but it is acknowledged that this may not be feasible and is beyond the scope of the current study.It would also have been interesting if the change in the outcome had been correlated with the change in hormone but this may be beyond the scope of the current analysis.**

*We agree with the reviewer that comparison with a cisgender control group would be a great study design. We added this as a suggestion for future research to our Discussion section (p. 19):*

“Future, larger studies in older transgender persons with a longer follow-up duration, different hormone regimes (i.e., other or no use of antiandrogens) and different administration types, preferably in comparison to cisgender controls, are necessary to expand our knowledge on this subject.”

*We also agree with the second recommendation of the reviewer, and therefore we had performed analyses with hormone concentration as a covariate, however there was no correlation between the height of the hormone concentration and the change in outcome (p. 13):*

“Lastly, higher hormone concentrations during treatment or different smoking status (at baseline) did not affect the results either.”

**Reviewer #2:** **This is an interesting report about the effect of hormone therapy on markers of inflammation and hemostasis in transgender persons. The findings were clear-cut; however, the manuscript has some problems.**

*We thank the reviewer for the time and effort reviewing our manuscript, and for the useful suggestions that* *helped us to improve our manuscript.*

**The keyword cardiovascular events should be revised.**

*This keyword was changed to cardiovascular risk.*

**Abstract. The result about the increment in platelet factor 4 (PF-4) in trans women is not mentioned.**

*This was added to the abstract (p. 2):*

“After 12 months, in trans women, systemic and endothelial inflammatory markers decreased (hs-CRP -66%, (95% CI -76 ; -53), VCAM-1 -12%, (95% CI -16 ; -8)), while platelet activation markers increased (PF-4 +17%, (95% CI 4 ; 32), β-thromboglobulin +13%, (95% CI 2 ; 24)).”

**Introduction. It is not clear why the authors measure leptin and adiponectin, they should explain in a little more detail why they chose these adipokines as adipose tissue markers.**

*We thank the reviewer for this comment. We clarified our interest in adipose tissue markers in the Introduction section (p. 4):*

“We selected a broad spectrum of markers associated with systemic, endothelial, or adipose tissue related inflammation”.

*We specifically selected leptin and adiponectin because they are known to be influenced by sex hormones (Auer et al., 2018, Elbers et al. 1997), and because they are associated with inflammation. We explained this more clearly in the following sentence (p. 6):*

“Adipose tissue specific marker leptin is associated with obesity and is a predictor of myocardial infarction (15, 16), while adiponectin has anti-inflammatory effects, like increasing insulin sensitivity (16, 17).”

**Materials and methods. The sentence in line 154 “Using EDTA or serum plasma samples” should be revised.**

*We now specified this sentence to enhance clarity (p. 7):*

“The above-mentioned markers were measured at the Netherlands Organization for Applied Scientific Research (Leiden, The Netherlands), using serum (for PF-4 and β-thromboglobulin) and EDTA (for all other markers) plasma samples and miniaturized biomarker assays.”

**Authors did not mention whether the dosage of the hormone therapy was adapted in some participants to reach adequate estradiol or testosterone concentrations as suggested by guidelines.** **The authors omit important characteristics of the study population as: blood pressure, weight, BMI, glucose levels, and lipid profile at baseline and after 3 and 12 months of hormone therapy.**

*We thank the reviewer for the valuable suggestions. We added blood pressure and BMI at 3 and 12 months to table 2 (p. 10):*

**Table 2. Hormone concentrations, body mass index and blood pressure at baseline, after 3 and 12 months of hormone therapy**

|  | Trans women | | | Trans men | | |
| --- | --- | --- | --- | --- | --- | --- |
|  | Visit (months) | | | | | |
|  | 0 | 3 | 12 | 0 | 3 | 12 |
| Estradiol level (pmol/L) | 101 (79-126) | 246 (120-342) | 258 (178-496) | 273 (168-442) | 184 (147-250) | 158 (116-204) |
| Testosterone level (nmol/L) | 18 (14-23) | 0.6 (0.5-0.8) | 0.7 (0.5-0.8) | 1.3 (1.0-1.7) | 27 (14-42) | 20 (15-30) |
| BMI (kg/m^2) | 23 (21-26) | 23 (21-26) | 24 (22-27) | 23 (21-30) | 23 (22-29) | 23 (22-28) |
| BP (mmHg) Systolic  Diastolic  MAP |  |  |  |  |  |  |
|  | 129 (123-138) | 123 (117-134) | 127 (117-135) | 122 (115-126) | 123 (117-130) | 122 (116-129) |
|  | 80 (73-88) | 78 (73-83) | 78 (73-84) | 77 (72-81) | 75 (70-82) | 76 (72-82) |
|  | 96 (91-101) | 93 (88-98) | 94 (89-100) | 92 (87-97) | 90 (86-98) | 92 (87-96) |

Data are presented as median (IQR). BMI, body mass index. BP, blood pressure. MAP, mean arterial pressure.

*Also, we added glucose, total cholesterol and triglycerides to table 1 (p. 9):*

**Table 1. Baseline characteristics of the study population**

|  | Trans women  (n = 48) | Trans men  (n = 47) |
| --- | --- | --- |
| Age (years) | 30 (24-39) | 23 (20-26) |
| BMI (kg/m^2) | 23 (21-26) | 23 (21-30) |
| Ever smoked (%) | 16 (33%) | 20 (43%) |
| Blood pressure (mmHg)  Systolic  Diastolic  MAP | 129 (121-137)  80 (76-87)  96 (91-101) | 122 (115-128)  77 (72-82)  92 (87-97) |
| Glucose (mmol/L) | 5.5 (5.1-5.7) | 5.1 (4.9-5.4) |
| Total cholesterol (mmol/L) | 4.3 (3.7-5.1) | 4.3 (3.9-4.9) |
| Triglycerides (mmol/L) | 0.9 (0.7-1.3) | 0.7 (0.5-1.0) |

Data are presented as median (IQR) for continuous data, and number and % for categorical
data. BMI, body mass index. MAP, mean arterial pressure. Participants with an underlying disease: diabetes mellitus (1), human immunodeficiency virus (2), pulmonal hypertension (1), ulcerative colitis (1).

**There are no data concerning the liver and renal function for the safety evaluation.**

*We thank the reviewer for this suggestion. While we acknowledge that safety of the hormone therapy is important, evaluating the safety of hormone therapy was not the aim of this study. Further, also note that we included patients that received routine clinical transgender care in the Netherlands, i.e. there was no experimental manipulation induced by our study. To clarify this issue, we have added this to our Study design section (p. 5):*

“Participants of the ENIGI-study are 18 years or older, diagnosed with gender dysphoria according to the revised fourth or fifth edition of the Diagnostic and Statistical Manual of Mental Disorders and receive routine clinical transgender care.”

**It is not specified if some participants had an underlying disease.**

*We now added this information to the caption of table 1, see above (p. 9).*

**In table 3 the authors should provide the p-values of all the comparisons.**

*P-values of all comparisons were added, see above (p. 11 and 12).*

**Figure 1 displays a reduction in IL-6, IL-8 and IL-22 in trans women at 12 months; these results are not mentioned in results section and are not discussed.**

*We now added the reduction in IL-6, IL-8 and IL-22 to the results (p. 13):*

“After 12 months of transdermal estradiol plus CPA, systemic and endothelial inflammatory marker concentrations decreased (hs-CRP -66%, (95% CI -76 ; -53), IL-6 -28%, (95% CI -7 ; -44), IL-8 -15%, (95% CI -2 ; -17), IL-22 -34%, (95% CI -7 ; -44), VCAM-1 -12%, (95% CI -16 ; -8)).”
